# A Biomechanical Model Correlating Shoulder Kinetics to Pain in Young Baseball Pitchers

**DOI:** 10.2478/v10078-012-0059-8

**Published:** 2012-10-23

**Authors:** David W. Keeley, Gretchen D. Oliver, Christopher P. Dougherty

**Affiliations:** 1 Department of Human Performance, Dance, and Recreation, New Mexico State University; Las Cruces, New Mexico.; 2 Department of Health Sciences, Kinesiology, Recreation, and Dance, University of Arkansas; Fayetteville, Arkansas.; 3 The Agility Center, Bentonville, Arkansas.

**Keywords:** baseball, kinetics, youth, injury

## Abstract

Previous work has postulated that shoulder pain may be associated with increases in both peak shoulder anterior force and peak shoulder proximal force. Unfortunately these relationships have yet to be quantified. Thus, the purpose of this study was to associate these kinetic values with reported shoulder pain in youth baseball pitchers. Nineteen healthy baseball pitchers participated in this study. Segment based reference systems and established calculations were utilized to identify peak shoulder anterior force and peak shoulder proximal force. A medical history questionnaire was utilized to identify shoulder pain. Following collection of these data, the strength of the relationships between both peak shoulder anterior force and peak shoulder proximal force and shoulder pain were analyzed. Although peak anterior force was not significantly correlated to shoulder pain, peak proximal force was. These results lead to the development of a single variable logistic regression model able to accurately predict 84.2% of all cases and 71.4% of shoulder pain cases. This model indicated that for every 1 N increase in peak proximal force, there was a corresponding 4.6% increase in the likelihood of shoulder pain. The magnitude of peak proximal force is both correlated to reported shoulder pain and capable of being used to accurately predict the likelihood of experiencing shoulder pain. It appears that those pitchers exhibiting high magnitudes of peak proximal force are significantly more likely to report experiencing shoulder pain than those who generate lower magnitudes of peak proximal force.

## Introduction

Within the little league baseball system, the Minor League Division is where young pitchers begin to participate in what are known as player pitch divisions (Little League® Online, 2010). In the Minor League Division, the primary focus is on developing the fundamentals of the game of baseball. For baseball pitchers, it is pitching mechanics (Steve Barr, Personal Communication, May, 2010). The development of proper pitching mechanics at this stage is vital since the most appropriate time to prevent injuries in pitchers’ is at the beginning of their careers (Fleisig et al., 1999; Nissen et al., 2007). Unfortunately, due to the complex nature of the pitching motion, the attempts of coaches to instill proper mechanics at the onset of pitchers’ careers has not resulted in decreasing injury rates in youth pitchers.

Without argument, the incidence of injury to youth baseball pitchers is on the rise and the primary injury is at the shoulder (Conn et al., 2003). In fact, previous examinations of injury rates in baseball players have reported that for every 10,000 athletic exposures there are 1.72 injuries experienced (Krajnik et al., 2010). Of the 91 total injuries reported, 43% were non-contact, 38% were suffered by pitchers, 24% were overuse in nature, and 48% were classified as muscle strain or tendonitis.

Although the previously stated statistics included analysis of both little league and high school baseball players, a recent survey examining injury rates in baseball players between the ages of 5 yr and 14 yr has indicated that 25% of the participants have experienced some type of injury while actively participating (Safekids online, May 2010). Another study of little league baseball injuries reported that between 1997 and 1999, nearly 25% of young baseball players in the United States suffered injuries during participation (Conn et al., 2003). In addition it was reported that the majority of these injuries were overuse with 26% involving the shoulder.

Within the available literature, kinetics within the shoulder have often been discussed as the underlying factors resulting in shoulder pain (Aguinaldo et al., 2007; Albright et al., 1978; Fleisig et al., 1995; Keeley et al., 2008; McFarland and Wasik, 1998). The two kinetic parameters most often discussed are, anterior force which peaks near the time of maximum shoulder external rotation, and proximal force which peaks near ball release (Fleisig et al., 1995; 1999; Keeley et al., 2008). Thus, the purpose of the current study was to associate peak shoulder anterior (PAF) force during the arm cocking phase and peak shoulder proximal force (PPF) during the arm acceleration phase with reported shoulder pain in youth baseball pitchers.

In the effort to achieve this, this study examined the relationships between PAF during arm cocking and PPF during arm acceleration and reported shoulder pain. It also investigated how these kinetic parameters regressed on the incidence of shoulder pain. It was hypothesized that shoulder pain would be associated with both PAF and PPF as these variables have previously been associated with shoulder injury (Fleisig et al., 1999; Oliver and Keeley, 2010). It was also hypothesized that the probability of young pitchers experiencing shoulder pain could be predicted from a linear combination of the two shoulder kinetics.

## Material and Methods

### Participants

Nineteen healthy youth baseball pitchers (mean age: 11.2 ± 1.7 years, body height: 142.6 ± 9 cm, body mass: 41.1 ± 6.3 kg) were recruited from the Northwest Arkansas area and participated in the current study. Testing procedures were approved by the University of Arkansas (Fayetteville, AR) Institutional Review Board and were similar to those identified in previous work (Oliver and Keeley, 2010a; Oliver and Keeley, 2010b). Prior to participation the approved procedures were explained to each participant and their parent(s) who provided consent.

### Procedures

Following the provision of consent, participants were prepared so that kinematic data could be collected using The MotionMonitorTM electromagnetic tracking system (Innovative Sports Training, Chicago IL). A series of 10 electromagnetic sensors were attached to the medial aspect of the torso (at C7) and pelvis (at S1), the distal/lateral aspect of both the throwing and non-throwing humerus and forearm, and the distal/lateral aspect of both the right and left thigh and shank (Myers et al., 2006). Subsequent to the attachment of the electromagnetic sensors, one additional sensor was attached to a wooden stylus and used to digitize the palpated position of the bony landmarks described in Table 1 (Meyers et al., 2006; Wu et al., 2005).

Following sensor attachment, pitchers were allowed to complete their pre-competition warm-up period in preparation for data collection. Test trails consisting of maximal effort fastball pitches toward a catcher located the regulation distance from an indoor pitching mound were conducted. For all test trials, pitches were delivered from the stretch position and those data from the fastest pitch passing through the strike-zone were selected for detailed analysis (Keeley et al., 2008; Guido et al., 2009; Sabick et al., 2004).

To collect data describing shoulder pain in the current study, a medical history questionnaire was utilized. This questionnaire, completed by both the participants and their parents/guardian collected information describing the following: 1) shoulder pain following throwing outing during the current competitive season; 2) shoulder pain frequency following a throwing outing; 3) level of relative shoulder pain on a scale of one through 10; and 4) loss of practice time or performance time due to shoulder pain.

Each of these variables was assessed for the most current competitive season

Collection of kinematic data was required to identify the phases of the pitch cycle. The collection rate for these data describing the position and orientation of electromagnetic sensors was set at 144 Hz. Raw data were independently filtered along each global axis using a 4th order Butterworth filter with a cutoff frequency of 13.4 Hz (Fleisig et al., 1999). Throwing kinematics for right handed participants were calculated using the standards and conventions for reporting joint motion recommended by the International Shoulder Group of the International Society of Biomechanics (Wu et al., 2002; 2005). The angle decomposition sequences used to describe the position and orientation of the torso, humerus, and forearm, as well as definitions of the movements they describe are shown in Table 2. Throwing kinematics for left handed pitchers were calculated by mirroring the world z axis. This allowed analysis of left handed pitchers to be described from a right hand point of view (Wu et al., 2002; 2005).

Throwing kinetics were calculated by modeling the torso and arm as four rigid links in series and connected by ball-and-socket joints (Fleisig et al., 1999; Keeley et al., 2008; Sabick et al., 2004b; Feltner and DaPena, 1986). Body segment masses and inertial parameters were obtained from previous literature and scaled to participant height and mass (Clauser et al., 1969; Hinrichs, 1990). Shoulder anterior force was defined as the anterior component of the resultant force acting along the anterior/posterior axis of the shoulder, while shoulder proximal force was defined as the component of the resultant force acting along the longitudinal axis of the shoulder (Keeley et al., 2008; Sabick et al., 2004a; Sabick et al., 2004b). Each of these forces was modeled using a convention that calculated the force applied by the torso to the proximal humerus and were normalized to percent bodyweight. It should be noted that internal kinetic model validation efforts revealed that the differences in estimated and observed forces were approximately 6.4% which is similar to those observed in previous research (Nesbit, 2005).

### Analyses

The data in the current study were analyzed using the Statistical Package for Social Sciences 15.0 (SPSS Inc, Chicago, IL). To identify the relationship between PAF, PPF, and shoulder pain, point-by-serial correlation coefficients were calculated. To address multicollinearity issues, the Pearson product moment correlation coefficient was calculated between PAF and PPF to assess the strength of the relationship between these parameters. Finally, logistic regression techniques were used to define the most efficient predictive model identifying the probability a baseball pitcher experiencing shoulder pain. In the current study, PAF and PPF were the independent variables and shoulder pain was the dependent variable..

## Results

The results of descriptive analyses are shown in Table 3. These analyses revealed that all model assumptions for both the correlation and logistic regression analyses were met. With regard to shoulder pain, 7 of the 19 participants (∼37%) reported experiencing shoulder pain at some point in their current competitive season.

The results of the point-by-serial correlation analyses revealed that although PAF was not significantly related to shoulder pain (r = −0.141, p = 0.565), PPF during arm acceleration was (r = 0.704, p = 0.001). Because only one of the kinetic variables was correlated with shoulder pain, only PPF was included in the logistic regression model. The results of this analysis are displayed in Table 4 and indicated that PPF was a significant predictor of shoulder pain (χ2 = 11.116, p = 0.001). The resulting beta coefficient (β = 0.046) indicated that for every 1N increase in PPF, there was a corresponding 4.6% increase in the likelihood of a pitcher reporting shoulder pain (ŷ = 0.046*PPF – 10.126, p = 0.016 for β). The result of classification analysis indicating the predictive capability of the model showed that the one variable model including peak proximal force was capable of correctly predicting 84.2% of all cases (pain / no pain) and 71.4% of pain cases in the sample.

## Discussion

It was the purpose of this study to associate PAF and PPF during pitching to reported shoulder pain. The results revealed while PAF during arm cocking was not a significant predictor of reported shoulder pain, PPF during arm acceleration was. This is important as both anterior force and proximal force have been postulated as contributing to possible injury mechanisms. The results of this study support the notion that proximal force during pitching may contribute to the incidence of shoulder pain, but also contradict this notion with regard to anterior force.

The increased odds ratio in shoulder pain that was observed with high levels of proximal force may be the result of specific pathologies within the shoulder, particularly within the glenoid labrum region. As proximal force at the shoulder (the result of the net forces applied by the torso to the upper extremity) increases, a corresponding increase in glenohumeral shear force may occur. This has the potential to result in micro trauma to the glenoid labrum. Also, this increased proximal force may result in additional glenoid labrum damage as the biceps contracts to both control elbow extension and stabilize the glenohumeral joint against distraction during arm acceleration. It has been shown that when the long head of the biceps brachii contracts forcefully, it has the propensity to lift the labrum off the glenoid (Andrews et al., 1985). The repeated lifting of the labrum may result in micro trauma to the labrum in young pitchers, eventually resulting in the development of SLAP lesions later in their careers (Snyder et al., 1990).

Unfortunately, the baseball pitching motion repeatedly places the throwing shoulder in highly unstable positions. As the function of the labrum is to deepen the fossa of the glenoid, providing increased stability to the glenohumeral joint, damage to this structure may ultimately decrease the ability of young pitchers to adequately stabilize the glenohumeral joint (Hall, 2007). Thus, it is important to identify young pitchers that may be at increased risk of glenoid labrum damage. Based on the results of this model, it is suggested that young pitchers who are reporting shoulder pain early in their career may be generating high magnitudes of proximal force within their shoulder. Because of this, intervention programs need to be developmental and implemented in attempt to curtail this injury predictor. Intervention strategies including incorporating torso control as well as scapular stabilization would provide a basis of developing a biomechanically efficient throwing shoulder. By increased control in the kinetic chain during the throwing motion, higher magnitudes of proximal shoulder force may potentially be offset through the better positioning of the humerus in relation to the scapula and torso thereby reducing the risk of injury.

## Figures and Tables

**Table 1:** Description of bony landmarks palpated and digitized in the current study

Bony Landmarks	Bony Process Palpated and Digitized
*Thorax*	
Seventh Cervical Vertebra (C7)	Most dorsal aspect of the spinous process
Eighth Thoracic Vertebra (T8)	Most dorsal aspect of the spinous process
Suprasternal Notch	Most cranial aspect of the sternum
*Humerus (Throwing and Non-throwing)*	
Medial Epicondyle	Most distal/medial aspect of the condyle
Lateral Epicondyle	Most distal/lateral aspect of the condyle
Center of Glenohumeral Rotation	Estimated*
*Forearm (Throwing and Non-throwing)*	
Radial Styloid Process	Most distal/lateral aspect of the radial styloid
Ulnar Styloid Process	Most distal/medial aspect of the ulnar styloid

The center of glenohumeral rotation (and subsequently the joint itself) was not digitized. It was estimated using a least squares algorithm for the point moving least during series of short rotational movements

**Table 2:** Descriptive statistics for peak shoulder kinetic parameters

Parameter	Min	Max	Mean	SD	SE	Skewness	Kurtosis
Anterior Force	−9.44	53.22	28.50	17.08	3.92	−0.16	−0.31
Proximal Force	130.59	296.14	201.59	48.68	11.17	0.48	−0.79

**Table 3:** Results of logistic regression analysis outlining the predictive relationship between P_PF_and shoulder pain

	B	S.E	Wald	df	p	EXP(B)	95% C.I for EXP(B)	

							Lower	Upper
							
P_PF_	0.046	0.019	5.762	1	0.016	1.047	1.009	1.088
Constant	−10.126	4.059	6.223	1	0.013	0.000		

Final logistic regression model equates to ŷ = 0.046^*^P_PF_– 10.126
